# Treatment and Management Experience of Idiopathic Granulomatous Mastitis in a Low-income Country

**DOI:** 10.1055/s-0043-1770089

**Published:** 2023-07-21

**Authors:** Pedro Antonio Llancari, Antonio Ortiz, Juan Becerra, Ricardo Muñoz, Christiam Valeriano, Rommy Helena Novoa

**Affiliations:** 1 Emergency Department, Instituto Nacional Materno Perinatal, Lima, Peru; 2 School of Medicine “San Fernando.” Universidad Nacional Mayor de San Marcos, Lima, Peru; 3 Gynecology Oncology and Breast Unit, Department of Gynecology, Instituto Nacional Materno Perinatal, Lima, Peru; 4 High-Risk Pregnancy Unit, Department of Obstetrics and Perinatology, Instituto Nacional Materno Perinatal, Lima, Peru

**Keywords:** idiopathic granulomatous mastitis, breast, surgery, corticosteroid, recurrence

## Abstract

**Objective** Reporting our experience of the management and treatment of Idiopathic granulomatous mastitis (IGM) in a low-income country by describing patients characteristics and therapy with emphasis on conservative surgical excision and postoperative care as the cornerstone of treatment.

**Methods** A retrospective cohort of women with histopathological diagnosis of IGM from 2014 to 2018 at Instituto Nacional Materno Perinatal in Lima, Peru. Patients' characteristics, clinical presentation, treatment, management, postoperative care, and follow-up were analyzed.

**Results** Thirty-eight patients with histopathological diagnosis of IGM were identified. Their average age was 35.9 years and 23 (60.5%) reported previous use of hormonal contraceptives. Nine (23.7%) patients had chronic mastitis with previous treatment. The time from the onset of symptoms to the first clinic consult was 5.1 months on average. Twenty-one (55.3%) patients had the lesion in the right breast, with a mean size of 6.9 cm. Conservative surgical excision was performed in all patients. Additionally, 86.8% required corticosteroids and 78.9% were treated with antibiotics. Complete remission was obtained at 141 days on average (range 44 to 292 days). Six (15.8%) women reported ipsilateral recurrence and 5 (13.2%), contralateral. The latency time was 25.5 months on average.

**Conclusion** The conservative surgical treatment demonstrated and close follow-up made for a high cure rate, but with recurrence similar to that reported in the literature. Use of gloves is an alternative to manage post operative wounds in a low-income country. The most frequent adverse effect was breast surgical scar.

## Introduction

Granulomatous mastitis (IGM) is a benign inflammation of the breast, of unknown etiology. 1 Several possible causes have been proposed, including immune reactions, infectious disease, and hormonal disturbances, though none has been fully tested. 2 This entity is a diagnosis of exclusion 3 ; thus, malignancy, infections such as tubercular mastitis (in countries with a high incidence of tuberculosis), 4 and systemic diseases that can induce its appearance must be ruled out. It occurs mainly in young multiparous women on average 35 years old, shortly after pregnancy. 5 There is no additional risk of subsequent breast cancer in these patients. 6

IGM is a therapeutic challenge, since more than 50% of reported cases are initially confused with other pathologies including breast cancer, 7 and a lot of patients have several courses of antibiotic and other treatments unnecessarily. Surgical treatment is not a widely accepted therapeutic option, although previous studies report it as an alternative in recurrent cases that did not respond to more conservative treatments. 8 Currently, there is no consensus to describe conservative surgical excision as a cornerstone intervention for idiopathic granulomatous mastitis as the best treatment for this pathology, and the frequency of recurrence is high. 1 7 Other studies have reported it as a successful alternative, with partial or total mastectomy offering a high percentage of cure compared with medical treatment options such as corticosteroids. 9 10 Accordingly, more effective and efficient therapeutic options should be sought in this pathology.

We report our experience of the management and treatment of IGM in a low-income country by describing patients characteristics and therapy with emphasis on conservative surgical excision and postoperative care as the cornerstone of treatment.

## Methods

This retrospective study was conducted at Instituto Nacional Materno Perinatal in Lima, Peru, one of the largest tertiary hospitals in South America fully dedicated to providing health care to women with gynecological and oncological problems and high-risk pregnancy and to their infants. Our institution is a reference center for all of Peru.

This cohort included all women with a histopathological diagnosis of IGM during the period from January 2014 to January 2018 who received treatment in our Gynecology, Oncology and Breast Unit. The data collection was performed by the study investigators through a review of the clinical history of the registered patients with histopathological diagnosis of IGM and later telephone communication with them.

A histological diagnosis of IGM was required on core needle biopsy or open surgical biopsy. Patients with the following characteristics were excluded: positive laboratory study for mammary tuberculosis, histopathological study with diagnosis of breast cancer or other associated breast pathology, immune disorders, mainly HIV-AIDS, pregnancy during the course of the disease. We collected data on sociodemographic characteristics, clinical presentation, different combinations of medical (use of antibiotics and/or steroids) and surgical management, postoperative care, follow-up, and outcomes. All patients had undergone a conservative surgical excision of the lesion for inflammatory granulomatous tissue.

Specific location of the lesion clinically and sonographically (avoiding further resection). Regional anesthesia is administered. A small incision is made, with drainage and removal of granulomatous tissue respecting the structure of the breast with ectoscopic negative margins with the aim of preserving the maximum volume of healthy tissue. After the excision, the cavity is washed with sodium chloride and surgical gloves are inserted whose function is to facilitate the exit as multiple lamellar drains, avoiding the accumulation of dead tissue, hemostasis, and premature adhesions. The open wound is subjected to daily dressing to achieve wound retraction, and surgical closure is performed in the outpatient consult as soon as the drainage of secretions is reduced to a minimum.

The antibiotics given were cephalosporins, quinolones, tetracyclines, or trimethoprim-sulfamethoxazole, for 7 to 21 days. Prednisone was given at a dose of 0.5 mg/kg a day for 2–3 weeks. After this, a tapering protocol began according to the clinical response, at the rate of 5 mg per week until it was reduced to the lowest dose, until 7 weeks approximately. We uses steroids depending on the dimensions of the lesion, the severity of the symptoms, and the patient's general health and personal treatment preferences. No patient received immunosuppressant therapy during the time of management in our institution.

The database was cleaned to eliminate repeated values, assign lost values, and recategorize variables for the final analysis. We performed a descriptive analysis to summarize the characteristics of the patients. The distribution of the absolute, relative, and accumulated frequencies of categorical variables was calculated. For numerical variables, summary measures were applied as averages and ranges. Statistical analysis was performed using Stata Statistical Software 14.0 (Stata Corp. 2015, College Station, TX, USA). We obtained ethical approval from the local Ethical Institutional Board (reference number: 169–2019-CIEI/INMP).

## Results

While 41 patients were identified with a histopathological diagnosis of IGM, three of them were excluded. Two patients had pregnancy of 10 and 18 weeks and one had a PCR positive for tuberculosis. We describe the characteristics of the 38 included patients in Table 1 . The average age of the women was 35.9 years. Twenty-three patients (60.5%) reported using hormonal contraceptives for at least six months, the most common being the injection (52.6%). Only 2 women reported a history of tuberculosis, one of them pulmonary and the other in the left breast. No patient reported smoking. A history of chronic mastitis was present in 9 patients (23.7%); 7 of them had it in the contralateral breast while 2 patients reported bilateral mastitis; all of them had received previous treatment, of whom four women had surgical treatment.

**Table 1:** Characteristics of patients with idiopathic granulomatous mastitis ( *n* = 38)

Characteristics	n(%)
Mean age in years (range)	35.9 (20 - 59)
Contraceptive methods	
Injection	20 (52.6)
Intrauterine device	2 (5.3)
Implant	1 (2.6)
None	15 (39.5)
Smoking	0 (0.0)
Previous tuberculosis	2 (5.26)
Previus mastitis	9 (23.7)
Previus treatment	9 (23.7)
Duration of symptoms in months (range)	5.1 (1–36)
Disease Laterality	
Right	21 (55.3)
Left	13 (34.2)
Bilateral	4 (10.5)
Size, cm (range)	6.9 (3–12)
Breast manifestations	
Mass	38 (100.0)
Swelling	34 (89.5)
Fistula	31 (81.6)
Nipple discharge	20 (55.6)
Imaging evaluation	
Ultrasaund	37 (97.4)
Mammogram	22 (57.9)

**Fig. 1:**
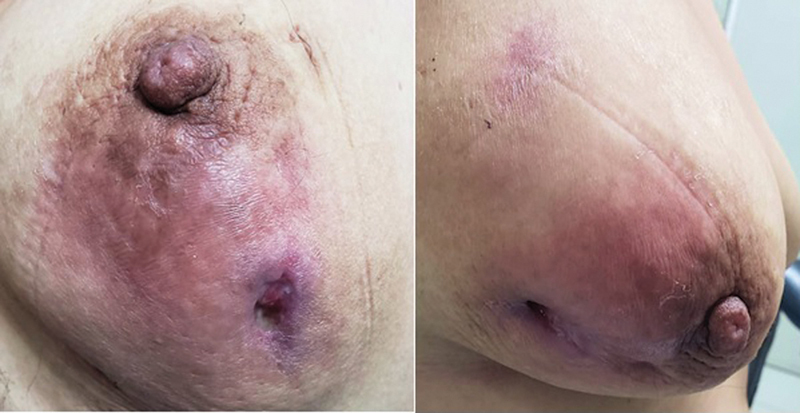
Erythema, phlogosis and ulceration with active suppuration in the lower quadrante. Gynecology Oncology and Breast Unit. INMP.

The time from the onset of symptoms to the first clinical visit was on average 5.1 months. Most of the patients (55.3%) had the lesion in the right breast, with average size of 6.9 cm. In the clinical evaluation, 34 (89.5%) patients had swelling; 31 (81.6%) presented fistulous trajectories, and 20 (55.6%) discharge from the nipple ( Fig. 1 ).

**Fig. 2:**
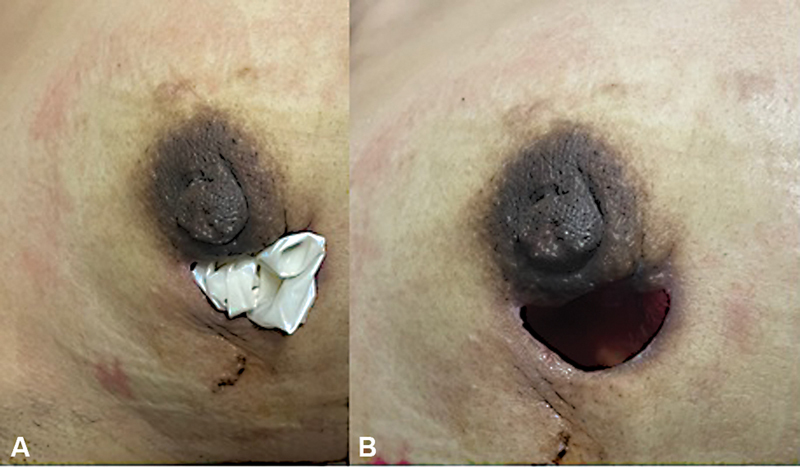
( **A** ) Surgical Gloves after surgery. ( **B** ) No adhesions or dischargue. Gynecology Oncology and Breast Unit. Instituto Nacional Materno Perinatal.

Among the 21 patients who had radiographic evaluation, the most frequent classification was BIRADS 0, in 16 patients. One patient was classified as BIRADS 4C, with subsequent confirmation of benignity of the lesion. Ultrasonography diagnosed chronic mastitis existed in 17 patients (44.7%), and signs of acute mastitis with abscesses and collections in 12 patients (31.6%). Only 21% of the patients had a biopsy before the start of treatment. The type of treatment is described in Table 2 . All the patients had conservative surgical excision of the lesion for IGM ( Fig. 2 ). Additional treatment was used in different combinations: corticosteroids and antibiotics were applied in 28 patients (73.7%), corticosteroids in 5 patients (13.2%), and only antibiotics in 2 patients (5.3%). While 86.8% of the patients received corticosteroids, 78.9% were administered an antibiotic cycle. The average number of healings during the open wound period among the patients was twenty.

**Table 2:** Treatment of mastitis granulomatous mastitis

Features	Patient (n)	Percent
Type of treatment		
Surgery + steroids + antibiotics	28	73.68
Surgery + steroids	5	13.16
Surgery	3	7.89
Surgery + antibiotics	2	5.26
Time from open surgical wound (days)	38	24.7 (13–50)
Time from diagnosis to resolution (days)	141	44 - 292
Recurrence		
Ipsilateral	6	15.8
Contralateral	5	13.2
Time of recurrence (months)	6	25.5 (3–84)

The resolution time for granulomatous mastitis was an average of 141 days, with a minimum of 44 days and a maximum of 292. Six (15.8%) women reported ipsilateral recurrence and 5 (13.2%), contralateral. The latency time was 25.5 months on average. The most frequent adverse effect reported was breast scar distortion. No side effects of corticosteroid, antibiotic, or NSAID therapy were reported.

## Discussion

This retrospective study showed the characteristics and type of treatment of 38 women with granulomatous mastitis treated in our Breast Unit. The conservative surgery treatment was performed in all patients and was associated with corticosteroids in 86.8%. Additionally, 78.9% received an antibiotic cycle. The resolution time was 141 days on average. Eleven patients (28.9%) had recurrence in 25.5 months on average.

Childbearing age between the 3rd and 4th decenniums is the most common in IGM diagnosis. In our study, the mean age of patients was 35.9 years. However, the literature reports 11 years as the youngest patient 11 and 83 years old as the oldest. 7 Contraceptive users are at increased risk of developing IGM. We reported 60% of patients with a history of using some hormonal contraceptive.

The pathogenesis and etiology of IGM is a not clear issue, but this disorder is characterized by granulomatous inflammation like many others diseases. 12 There are many agents and diseases related, such as local irritants, viruses, bacteria, mycotic and parasitic infections, diabetes mellitus, smoking, and systemic immune abnormalities. 10 13 Any etiological factors could permit damage to the ductal epithelium, allowing luminal secretion to leak into the lobular connective tissue, and an autoimmune reaction to these extravasated secretions was hypothesized. 11 However, the trigger in the development of this epithelial damage remains unknown. 2

Mastitis tuberculosis should be a differential diagnosis for IGM, even more so in Peru that has 14% of the estimated cases of tuberculosis in the America Region. 14 Mammary tuberculosis has been estimated to be 0.1% of breast lesions examined histologically. Breast tuberculosis is paucibacillary, and routine diagnostic tests such as microscopy and culture and nucleic acid amplification tests such as PCR techniques do not have the same diagnostic utility as they do in pulmonary tuberculosis. 15 We reported only 2 patients with previous lung tuberculosis infection, but in none of them was TB found. Pinto et al reported 28 patients with chronic tuberculous granulomatous mastitis in a Peruvian hospital; however, only 3 patients in that cohort had PCR positive, and 2 of them, BK culture. 16

The most described clinical presentation was mass (80 -100%), 17 18 fistula (16–52%), 19 and inflammation of erythema (11%). 10 20 In this study all patients had a breast mass associated with phlogosis, nipple discharge, or fistulas. Although bilateral involvement has rarely been reported, 4 (10%) patients had this characteristic. In the other patients, there was predominance in the right breast. IGM remains a diagnostic challenge for clinicians. The delay in diagnosis is around 5.1 months on average, similar to what a systematic review of 70 articles with 3060 patients found. 21

The diagnosis of IGM is one of exclusion and should be based on a multidisciplinary approach. Thus, all infectious and noninfectious causes of granulomatous inflammation must be excluded. 11 The gold standard for definitive diagnosis is core breast biopsy. 22 Image studies could help differentiate malignant lesions and thus avoid adverse effects of aggressive treatments when cancer is suspected. 23 Thus, ultrasound may show the number of masses and the heterogeneity with diffuse parenchymal edema, fluid in fat planes, and abscess. 8 Mammography is used to identify suspicious for malignancy with ill-defined focal densities and spiculated pattern. Another technique is Magnetic Resonance Imaging (MRI), whose findings include parenchymal enhancement, with asymmetrical signal intensity changes. However, the heterogeneous spectrum of presentation of IGM results in all images' findings being inconclusive for differentiating benign from malignant disease. 24 In our patients, only 21% had core breast biopsy previous to surgical management and almost 100% had ultrasound as the study protocol. However, only 55% had access to mammography, and no patient had MRI. These data reflect that the diagnosis in low-income countries such as Peru has such difficulties. Thus, we trust in clinic presentation and keeping a high index of clinical suspicion in cases of mastitis not responsive to antibiotics or a lot of other failed treatments.

Therapies for IGM included surgery (mastectomy, excision, and drainage), drug therapies (antibiotics, corticosteroids, immunosuppressive drugs, anti-inflammatory drugs, and others), and simple observation. However, the optimal treatment of IGM is still controversial due to varying degrees of success, and recurrence rates remain as high as 50%. 7 21 25 Moreover, the management of this pathology is based on observational studies, most of which were retrospective, with only a few prospective ones. 7 20 No clinical trials of treatment of this pathology are reported, and therefore the quality of the data are not optimal.

Bouton in 2015 followed up 37 cases of IGM, of which 27 cases resolved spontaneously without treatment; but the authors takes into account that the evolution of the disease is long and the quality of life was affected. 26 There are reports of up to 59% women who received single or multiple courses of antibiotics without any response. 8 Corticosteroids are usually used in IGM; methylprednisolone, 17 prednisolone, and prednisone 27 have been used with different schemes. Although Pandey et al reported 80% complete resolution of the disease, the median time was 159 days. 8 Methotrexate is an alternative treatment; it is an immunosuppressor agent used in IGM patients with favorable results, and it is usually combined with corticosteroids when the patient does not consider a surgical option at first. 28 29 30 Haddad et al reported complete remission of moderate to severe IGM after treatment with methotrexate. However, almost half of these patients received prednisone as a combinate regimen, and 17.6% experienced relapse of the disease. 28

Surgery is the most widely used technique to reduce symptoms quickly 17 ; moreover, recurrence, fistula formation (4.7 to 30%), and infection are documented complications after these procedures. 2 20 31 Lai et al consider that surgical treatment should be applied only for patients with refractory or recurrent mastitis. Thus, the frequency of recurrence is high in all types of surgery except for total mastectomy. 9 32 Wang et al in 2020 reported a novel technique to avoid bigger scars, of using indwelling hoses and surgical inlets. 17 A systematic review reported in 2017 that treatment with corticosteroids provides significant regression of inflammatory lesions, allowing the possibility of conservative surgery that could decrease the healing time as well as the frequency of recurrence in the patient and improve her quality of life. 33

In our Breast Unit, aggressive and definitive treatment of mastitis was performed by conservative surgical excision associated with corticosteroids, with some complementary treatments such as antibiotic therapy and analgesics. The resolution time reported in our study was an average of 141 days. In the literature, it is reported as approximately up to 159 days 8 with various treatment options, and a total cure for between 42 to 93% of patients using a combination of only conservative surgery and corticosteroids. 8 32 Recurrence is variable, at 16 to 50% among patients with strict follow-up and without treatment 7 ; however, we report a recurrence of 29%. It is a suitable option for treatment and management of IGM in our low-income country scenario. However, as the sample size of this cohort was limited and a longer follow-up period is necessary, we should be more cautious when making recommendations.

It is a retrospective cohort study, based on review of medical records and phone calls; the analysis could have biases of memory or information bias. The main form of diagnosis was based on clinic manifestation and ultrasound features; some patients had biopsy before surgery, but all patients had mastitis histology confirmation. There is no protocol for management in our country, so there are several ways to approach management. This analysis is the largest Peruvian case series of patients with granulomatous mastitis with a combination of conservative surgical treatment and corticosteroids. All our patients had histopathological diagnosis of granulomatous mastitis, and all of them had very close follow-up until resolution. This study allowed building a local management protocol for this pathology because our institution is a national reference center. Larger observational studies and clinical trials are required to allow statistical comparison of the types of treatment.

## Conclusion

Granulomatous mastitis is a rare, benign breast disease but locally aggressive, which decreases the quality of life of the patient and that still does not have a standardized treatment. Conservative surgical treatment associated with corticosteroids and close follow-up until resolution are demonstrated to be a highly effective cure, but with recurrence rates similar to those reported in the literature. The most important adverse effect of the type of treatment applied in the study was breast scarring.
